# Understanding of the Electrochemical Behavior of Lithium at Bilayer-Patched Epitaxial Graphene/4H-SiC

**DOI:** 10.3390/nano12132229

**Published:** 2022-06-29

**Authors:** Ivan Shtepliuk, Mikhail Vagin, Ziyauddin Khan, Alexei A. Zakharov, Tihomir Iakimov, Filippo Giannazzo, Ivan G. Ivanov, Rositsa Yakimova

**Affiliations:** 1 Department of Physics, Chemistry and Biology, Linköping University, SE-58183 Linköping, Sweden; tihomir.iakimov@liu.se (T.I.); ivan.gueorguiev.ivanov@liu.se (I.G.I.); rositsa.yakimova@liu.se (R.Y.); 2 Laboratory of Organic Electronics, Department of Science and Technology, Linköping University, SE-60174 Norrköping, Sweden; mikhail.vagin@liu.se (M.V.); ziyauddin.khan@liu.se (Z.K.); 3 MAX IV Laboratory, Lund University, Fotongatan 2, SE-22484 Lund, Sweden; alexei.zakharov@maxiv.lu.se; 4 CNR-IMM, Strada VIII, 5, 95121 Catania, Italy; filippo.giannazzo@imm.cnr.it

**Keywords:** lithium, SiC, epitaxial graphene, cyclic voltammetry, Raman, chronoamperometry

## Abstract

Novel two-dimensional materials (2DMs) with balanced electrical conductivity and lithium (Li) storage capacity are desirable for next-generation rechargeable batteries as they may serve as high-performance anodes, improving output battery characteristics. Gaining an advanced understanding of the electrochemical behavior of lithium at the electrode surface and the changes in interior structure of 2DM-based electrodes caused by lithiation is a key component in the long-term process of the implementation of new electrodes into to a realistic device. Here, we showcase the advantages of bilayer-patched epitaxial graphene on 4H-SiC (0001) as a possible anode material in lithium-ion batteries. The presence of bilayer graphene patches is beneficial for the overall lithiation process because it results in enhanced quantum capacitance of the electrode and provides extra intercalation paths. By performing cyclic voltammetry and chronoamperometry measurements, we shed light on the redox behavior of lithium at the bilayer-patched epitaxial graphene electrode and find that the early-stage growth of lithium is governed by the instantaneous nucleation mechanism. The results also demonstrate the fast lithium-ion transport (~4.7–5.6 × 10^−7^ cm^2^∙s^−1^) to the bilayer-patched epitaxial graphene electrode. Raman measurements complemented by in-depth statistical analysis and density functional theory calculations enable us to comprehend the lithiation effect on the properties of bilayer-patched epitaxial graphene and ascribe the lithium intercalation-induced Raman *G* peak splitting to the disparity between graphene layers. The current results are helpful for further advancement of the design of graphene-based electrodes with targeted performance.

## 1. Introduction

Rechargeable and portable lithium-ion batteries (LIBs) are currently at the forefront of device research due to their significant potential as a high-performance power source in sustainable electronics [1,2,3,4,5] that is certified by the 2019 Nobel Prize in Chemistry [6]. The cutting-edge LIB technologies are successfully commercialized and well documented in numerous review papers [7,8,9,10,11,12,13,14,15]. However, demands of humankind for more powerful and cheaper energy storage devices have shown a rising trend from year to year and are in line with market growth for electrically driven cars, smartphones, and computers. In this regard, the development of next-generation energy storage devices possessing enhanced performance requires both the incorporation of new LIB components, namely electrodes, to existing technologies and a deep understanding of the interfacial chemistry of electrode materials interacting with lithium species. Many research groups have investigated the possibilities of replacing the most popular anode material, specifically graphite, by more cost-effective analogs [16,17,18,19,20,21]. Although graphite has a good electrical conductivity, its maximal theoretical specific capacity is limited to 372 mAh/g, which results in a low power density of LIBs [22]. Due to its higher specific capacity (4200 mAh/g) caused by the large ability to accommodate lithium ions [23], silicon (Si) is regarded as an alternative to graphite. Nevertheless, its poor reversibility (causing high charge-transfer resistance) and 300% volume expansion (causing disintegration of the electrode [24]) hinder the credibility of this material, and, as a result, the choice of an optimal anode material possessing balanced electronic conductivity and storage capacity is still a great challenge nowadays.

The integration of silicon and carbon technologies can enhance the lithium storage performance [25,26,27,28,29,30,31,32] and, therefore, is regarded as a promising approach to design a LIB anode with desirable properties. Particularly, it was shown that silicon-carbon composites and related hybrid materials can be used for the fabrication of high-capacity electrodes for lithium-ion batteries with excellent cycling life [25,27]. The mechanism behind the enhancement of the energy storage parameters of such Si-C hybrid anodes is governed by synergetic effects originating from the unique individual properties of each component in respect of lithiation. On the one hand, silicon has a large binding ability to lithium, providing high capacity. On the other hand, despite carbon possessing a smaller specific capacity compared to silicon, it undergoes no significant structural changes and volume expansion, acting as an effective supporting architecture and electron conducting pathway [33,34]. From a technological point of view, the most logical way to capitalize on the benefits of Si-C hybridization is to utilize silicon carbide (SiC) for energy storage. However, SiC itself is inert with respect to lithiation [35,36]. Fortunately, graphenization is believed to activate the nominally inert SiC matrix, thereby enabling lithium insertion [37,38,39,40,41,42]. As documented in earlier studies [43], the successful lithium intercalation beneath the first graphene and buffer layer on SiC was achieved at a heating temperature of about 350 °C and was confirmed by the appearance of two π-bands. Another group showed that the formation of highly conductive graphene on 6H- or 4H-SiC causes an enhancement of the Li ion capacity to the value of 670 mAhg^−1^, which is much higher than the graphite capacity limit [37]. Furthermore, it is well known that during lithiation, a solid electrolyte interphase (SEI) is formed at the anode interface, prohibiting lithium intercalation, or even causing irreversible capacity changes of the whole system. In this case, graphene on SiC may allow the formation of a textured SEI (for instance, LiF) with a preferential orientation, favoring lithium diffusion inside the electrode [42]. A significant improvement of the performance of lithium-ion batteries (reversible capacity of 1044 mAhg^−1^ at 100 mAg^−1^) by using a graphene/SiC composite as an anode has been recently demonstrated by Xinli et al. [38]. Another interesting observation is that the graphene-encapsulated and SiC-reinforced silicon nanowires can also provide a high lithium storage capacity of 1650 mAhg^−1^ [39]. All these examples indicate that the specific capacity for the LIB anode may be meaningfully controlled through adjusting the graphene quality and fabrication conditions.

A much better storage capacity of graphenized SiC compared to graphite cannot originate entirely from the graphene presence, hinting at a significant role of the Si surface in lithium binding. Considering the technological controllability of the graphenization of SiC, there is plenty of room for improvement of the capacity through smart design of the anode material toward the most favorable structural and electrochemical properties. This indicates that two-dimensional carbons supported on SiC, represented by epitaxial graphene, have a significant potential as novel anode materials in LIBs. In this context, a clear understanding of the room-temperature lithiation of epigraphene on SiC is central to realizing future LIBs.

Much effort has been devoted to performing lithium intercalation into epitaxial graphene [44,45,46,47,48,49,50,51,52,53,54,55] and to elucidating its physics nature [56,57,58,59,60,61,62]. In most cases, lithium intercalation has been achieved through room-temperature deposition of lithium species on epitaxial graphene from an alkali-metal getter source with subsequent thermal annealing at a selected temperature. One particularly interesting approach of lithium intercalation techniques comprises the deposition of slow Li^+^ ions of energy 5 eV by using a low-energy alkali metal ion gun followed by heating [63]. It was argued that the small pore size of graphene makes the direct penetration of lithium species through the hollow site (center of the hexagonal ring of the honeycomb structure) thermodynamically unfavorable (due to high intercalation barrier) and, therefore, lithium intercalation mainly occurs from the side of the SiC steps or through grain boundaries/intrinsic vacancy defects. These reports showed the principal possibility of lithium penetration through the graphene membrane, but the intercalation mechanisms as well as the behavior of lithium at early stages of the intercalation process are still not fully understood. Further development of lithium-ion batteries exploiting epitaxial graphene on SiC as an anode unambiguously demands the attainment of more reliable control of the lithiation process.

Inspired by a recent work on the electrochemical intercalation of graphene/h-BN van der Waals heterostructures [64], in the present work, we apply the lithium electrodeposition approach (never studied before for such a system) to explore the electrochemical activity of lithium on bilayer-patched monolayer graphene on SiC (BLPMLG/SiC) grown by thermal decomposition of the 4H-SiC substrate in an argon atmosphere. The advantage of such an electrode material lies in the hypothesis that the edges of bilayer graphene inclusions (patches) are efficient intercalation pathways. Therefore, bilayer graphene offers extra intercalation sites (between two C layers) to accommodate lithium species, which is conducive to provide high specific capacity. Concomitantly, bilayer graphene patches usually start to form at step edges [65,66], which also function as channels for lithium intercalation [44]. Therefore, the overgrowth of bilayer graphene may improve the kinetic performance of the epitaxial graphene/SiC electrodes. By performing in-depth Raman mapping followed by statistical analysis and density functional theory calculations, we shed light on the nature of the structural changes of the BLPMLG/SiC induced by electrochemical lithiation. Chronoamperometry and cyclic voltammetry measurements allowed us to understand the initial stages of lithium deposition and gain insights into the lithium electrochemistry at the BLPMLG/SiC interface.

## 2. Materials and Methods

Bilayer-patched epitaxial graphene was prepared through high-temperature thermal decomposition of the Si-face 4H-SiC substrate (which was cut out from the semi-insulating on-axis 4H-SiC wafer) in a vertical radiofrequency-heated furnace under patent-protected growth conditions [67]. The qualities of as-prepared bilayer-patched epitaxial graphene and a fraction of the bilayer in the samples were verified by using Raman mapping analysis and optical reflectance mapping, respectively. We employed a micro-Raman setup based on a monochromator (Jobin-Yvon, model HR460) equipped with a CCD (charge-coupled device) camera and diode-pumped solid-state laser as an excitation source. The laser wavelength was chosen to be 532 nm. The laser power was set to be 1 mW. The Raman spectra were taken using a large numerical aperture (NA = 0.95) 100× micro-objective lens. The spectral resolution of the system was ∼5.5 cm^−1^. Optical reflectance mapping was performed using the same micro-Raman setup [68]. To discriminate bilayer graphene regions from monolayer graphene regions, we conducted atomic force microscopy (AFM) measurements (morphology and phase imaging) with Si probes using the DI3100 equipment with a Nanoscope V controller. Afterward, local mapping of the differential capacitance signal (dC/dV) was carried out on the same sample regions using the same equipment with the scanning capacitance microscopy (SCM) module and Pt-coated Si tips. The SCM maps were collected by a high-sensitivity capacitance sensor connected to the tip, while applying an AC modulating bias with an amplitude of 0.5 V and 100 kHz frequency to the sample backside. Under these measuring conditions, the probed capacitance signal is associated with the graphene quantum capacitance C_Q_ [69].

Electrochemical measurements were undertaken with an O-ring-type three-electrode electrochemical cell [70,71] composed of BLPMLG/SiC as a working electrode, Ag/AgCl as a reference electrode, and platinum wire as a counter electrode. A computer-controlled potentiostat (Autolab, EcoChemie, Metrohm, Utrecht, The Netherlands) was utilized to perform all room-temperature electrochemical measurements. To elucidate the redox behavior of lithium at BLPMLG/SiC, we initially carried out cyclic voltammetry measurements (at a scan rate of 20 mV∙s) within a potential range from −3.5 V to −2 V vs. the Ag/AgCl reference electrode in a PC (propylene carbonate)|0.5 M LiClO_4_ (lithium perchlorate) electrolyte in a glove box. To explore lithium kinetics at bilayer-patched epitaxial graphene, we then conducted chronoamperometry measurements and recorded the current–time transients during the early stages of lithium electrodeposition on the BLPMLG/SiC electrode. Finally, lithium electroplating was performed at two different potentials: −4 V and −5 V. The corresponding lithiated BLPMLG/SiC electrodes were further subjected to detailed Raman study to investigate the lithium-induced effects at the graphene–SiC interface, including possible intercalation phenomena and defects generation in graphene. X-ray photoemission electron microscopy (XPEEM) and selected-area XPS (micro-XPS) were performed at the synchrotron radiation facility at MAX IV (Lund, Sweden) using the MAXPEEM beamline housing an ACLEEM microscope (Elmitec GmbH). Photoelectron spectromicroscopy was employed to ensure the presence of a Li^0^ and Li-containing SEI at the graphene electrode surface.

**Figure 1:**
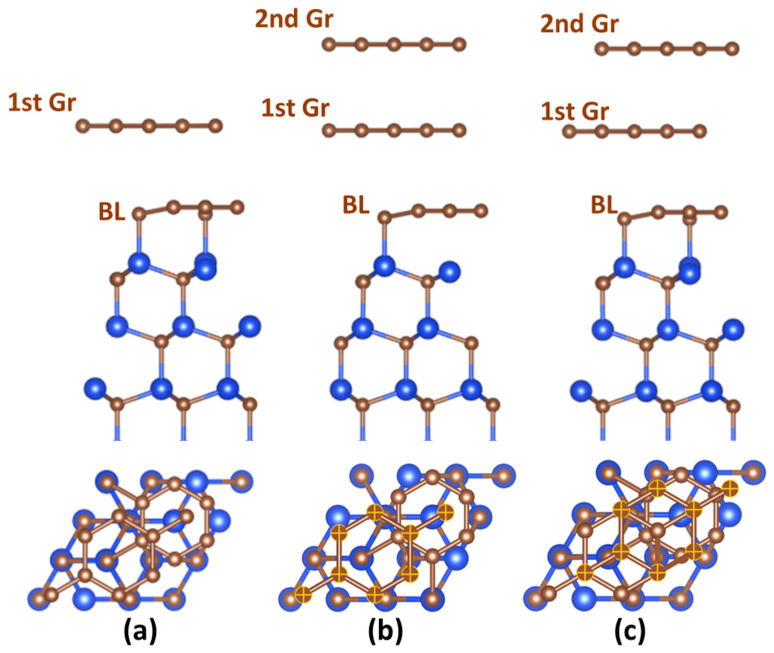
(Side and top views) Optimized structures of MLG/SiC (**a**), AA-BLG/SiC (**b**), and AB-BLG/SiC (**c**) electrodes. BL designates the buffer layer. Blue and brown balls represent silicon and carbon atoms, respectively, while yellow crosses denote the carbon atoms belonging to the second graphene layer. For the sake of convenience, we demonstrate three Si-C bilayers of 4H-SiC layers, although the original structures include nine Si-C bilayers. This is to avoid the substrate thickness effect on lithium adsorption and intercalation.

All density functional theory (DFT) calculations were executed with Siesta-4.1-b4 code [72] using the GGA-PBE functional [73] and double-ζ-polarized (DZP) basis set with an energy shift of 200 meV. In our previous investigations [74], we showed that the DZP basis set is good enough to reproduce the fundamentally important properties of the EG/SiC system and to predict the adsorption behavior of selected metals on EG/SiC that is consistent with experimental findings [75,76]. The density matrix tolerance in the self-consistent-field (SCF) cycle was 1 × 10^−5^, while the force tolerance in coordinate optimization was set to be 0.02 eV/Å. The Brillouin zone was sampled using a 9 × 9 × 1 Monkhorst–Pack *k* point grid. Norm-conserving Troullier–Martins pseudopotentials for C, Si, H, and lithium were generated by using ATOM code [77]. A BLPMLG/SiC electrode interface exposed to lithiation was modeled by three separate slabs: monolayer graphene on 4H-SiC (which is the core structure for the next two ones), AA-stacked bilayer graphene on 4H-SiC, and AB-stacked bilayer graphene on 4H-SiC. For the sake of convenience, these structures are referred to as MLG/SiC, AA-BLG/SiC, and AB-BLG/SiC, respectively. To construct the core structure of MLG/SiC, we utilized a 2 × 2 hexagonal graphene lattice on a √3 × √3R30° surface-reconstructed 4H-SiC (0001), which is a commonly used model of epitaxial graphene on 4H-SiC (0001) [78,79,80]. The optimized structures for all considered electrode interfaces before lithiation are depicted in Figure 1.

Further, we study the process of lithium intercalation into the electrode interface using the climbing image nudged-elastic band (CI-NEB) method. CI-NEB calculation was performed using SIESTA-LUA along with a flos library [81]. The image-dependent pair potential (IDPP) method was employed to generate the images of the system between the initial (Li-adsorbed electrode) and final (Li-intercalated electrode) configuration in an Atomic Simulation Environment (ASE) combined with Python library sisl.

## 3. Results and Discussion

### 3.1. Quality of Bilayer-Patched Epitaxial Graphene on 4H-SiC

**Figure 2:**
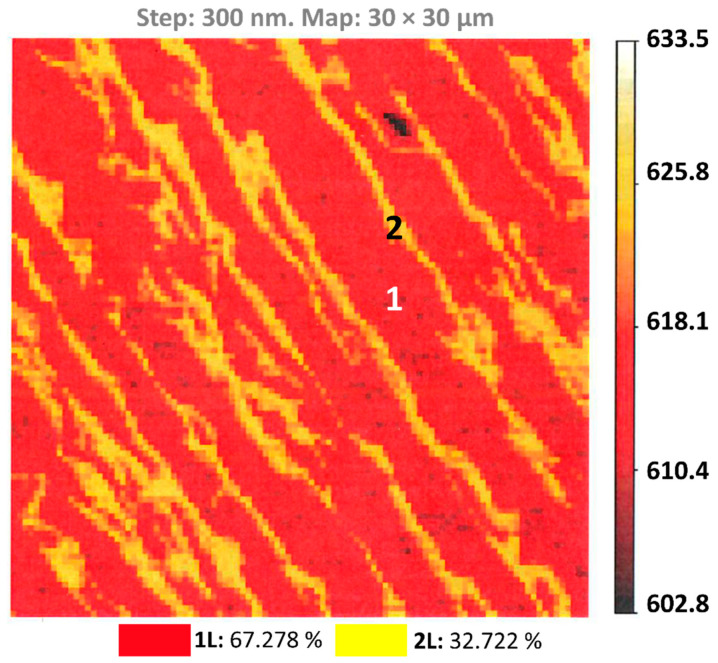
The optical reflectance map showing the co-existence of monolayer (marked by 1) and bilayer (marked by 2) epitaxial graphene regions on a 30 × 30 μm^2^ area.

Optical reflectance mapping (Figure 2) showed that the grown epitaxial graphene reproduces very well the terrace-stepped morphology of the 4H-SiC substrate. The majority of the surface (67.3%) is covered by a monolayer graphene, while bilayer graphene occupies ~32.7% of the total area (yellow-colored, narrow, parallel, nearly equidistant strips). It is important to emphasize that monolayer graphene predominantly appears at the wide terraces, while the bilayer graphene starts to form at the step edges.

**Figure 3:**
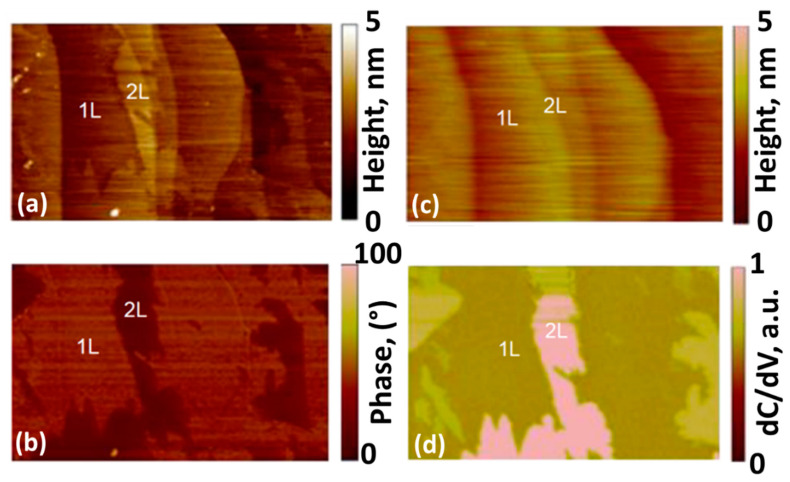
Tapping-mode AFM morphology (**a**) and phase (**b**) images of bilayer-patched epitaxial graphene on 4H-SiC. (**c**) Contact-mode AFM morphology of the same sample. (**d**) Derivative of the capacitance (dC/dV) between tip and sample.

To strengthen the evidence on the presence of bilayer graphene patches, we then performed AFM measurements (Figure 3). From the comparison between the tapping mode morphology and phase (Figure 3a,b), one can deduce quite easily the presence of monolayer and bilayer graphene regions, with the bilayer patches showing a lower phase signal in Figure 3b. On the same area, we performed scanning capacitance microscopy measurements. In Figure 3c,d, the morphology (in contact mode) and the derivative of the capacitance (dC/dV) are demonstrated. In the bilayer graphene regions, a significantly larger differential capacitance signal is measured with respect to monolayer areas. Indeed, these differences can be ascribed to the different quantum capacitance (C_Q_) values of monolayer and bilayer graphene at the same tip/sample bias. As the quantum capacitance is directly linked to the electronic density of states, it tends to increase with the increase in graphene thickness [82]. These results additionally enable the discrimination of the bilayer graphene regions from the monolayer graphene regions. As a high quantum capacitance of graphene correlates with a stronger Li binding [83], the presence of bilayer graphene patches can be useful for the design of higher-capacity LIB anodes. One more positive aspect of bilayer patches is that the bilayer graphene is more beneficial than monolayer graphene for providing the growth of a stable and highly conductive SEI [84].

**Figure 4:**
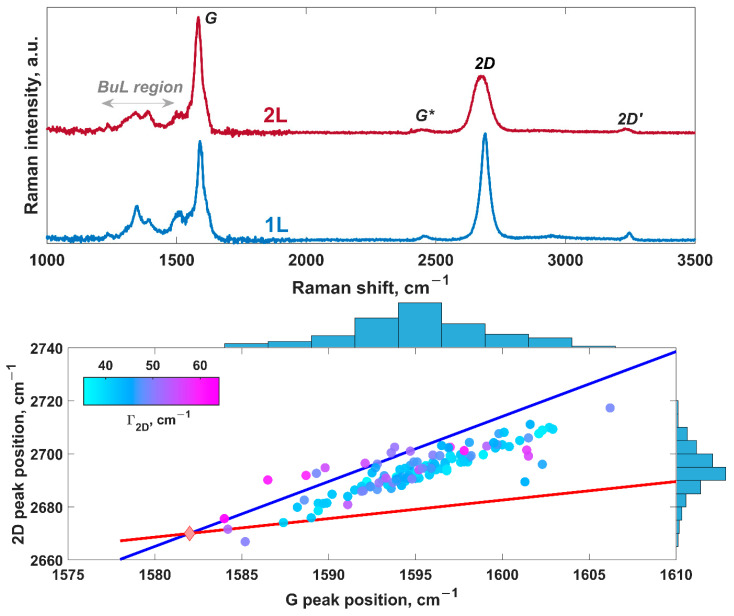
(**Top panel**) Raman spectra of monolayer (**bottom**) and bilayer (**top**) epitaxial graphene. (**Bottom panel**) 2*D* peak position vs. *G* peak position for bilayer-patched epitaxial graphene color-coded by FWHM (full width at half maximum, Γ_2*D*_) of the 2*D* peak. Corresponding histograms of 2*D* peak and *G* peak positions are also demonstrated. The reddish diamond designated by NP represents the neutrality point of free-standing graphene, without doping and strain effects. The blue and the red solid lines correspond to the so-called strain and doping lines for free-standing graphene.

To appraise the structural quality of the epitaxial graphene samples before lithiation, we also performed Raman mapping analysis. Two types of Raman spectra were recorded (Figure 4, top panel). Both spectra include characteristic *G* and 2*D* bands, as well as weak spectral features related to the buffer layer, inter-valley scattering (G*) band, and 2*D*’ peak. No *D* peak is seen in both spectra, pointing out the high structural quality of the graphene and small defect density. This enables us to exclude the defects as possible adsorption sites and intercalation paths during the lithiation process. Usually, the analysis of the interrelationships between characteristic peaks’ parameters provides valuable information on the number of graphene layers. For the exfoliated graphene, the 2*D*/*G* peak intensity ratio is an important descriptor of the graphene thickness. However, this is not always helpful in the case of epitaxial graphene on SiC. A more reliable way to distinguish bilayer from monolayer epitaxial graphene is to compare FWHM values of the 2*D* peak [65,85,86]. It is believed that the 2*D* peak with FWHM values ranging from 30 cm^−1^ to 40 cm^−1^ can be attributed to monolayer epitaxial graphene on SiC, while bilayer graphene typically demonstrates broader 2*D* peaks with FWHM ~45 cm^−1^ to 65 cm^−1^ [65,86]. Bearing this in mind, we can ascribe the demonstrated spectra in Figure 4 to monolayer and bilayer graphene, respectively. Assuming that the lower limit of the FWHM of 2*D* in the case of bilayer graphene is 45 cm^−1^, we also estimated the percentage of Raman spectra with 2*D* peaks having FWHM values higher than 45 cm^−1^. It is found to be 35.5% (also see bottom panel of Figure 4), which is in good agreement with the optical reflectance mapping results. From Figure 4 (bottom panel), it is also seen that the data points in 2*D*–*G* space are aligned to the strain line with a slope of 2.4. This points to the common statement that the epitaxial graphene is *n*-doped and compressively strained [74].

### 3.2. Cyclic Voltammetry Measurements

**Figure 5:**
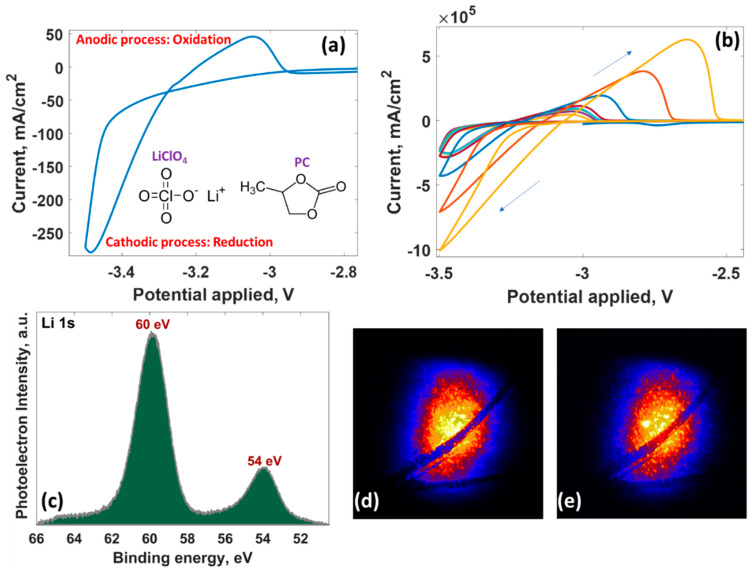
Cyclic voltametric data for the reduction/oxidation of Li^+^/Li^0^ at BLPMLG/SiC electrode in PC|LiClO_4_ electrolyte after first cycle (**a**) and after 11 cycles (**b**). (**c**) XPS spectrum of Li 1 s core-level peak, background subtracted. Elemental XPEEM contrast images of the lithiated BLPMLG/SiC electrode BLPMLG/SiC for Li 1 s peak at (**d**) 60 eV and (**e**) 54 eV. Field of view, 50 μm. The size of the photon beam is 15(h) × 20(v) µm^2^.

The lithium plating/stripping behavior on epitaxial graphene was then scrutinized by performing cyclic voltammetry measurements. It was found that metallic lithium deposition (cathodic process) on the electrode occurs at a potential of around −3.4 V, while the reverse reaction (anodic process) is observed at −3.0 V (Figure 5a). The peak-to-peak separation is estimated to be ~473 mV, indicating that the Li-related redox process at the surface of the BLPMLG/SiC electrode is an electrochemically quasi-reversible process. The repetitive scans demonstrate an increase in current during successive cycles. After 11 cycles, an increased response for lithium plating and stripping is observed (Figure 5b), suggesting a negligible role of the solid electrolyte interphase layer (SEI layer) in lithium kinetics at the interface as the bilayer-patched epitaxial graphene can accept more and more lithium species. Micro-XPS and XPEEM measurements (Figure 5c–e) after lithium plating at −5 V during 1 min showed that lithium is almost everywhere over the sample. Lithium on the BLPMLG/SiC electrode has two chemical states. The peak at 54 eV comes from metallic lithium, while the peak at 60 eV belongs to an SEI composed of lithium compounds (Li_2_O, LiOH, Li_2_CO_3_, etc.) [87].

### 3.3. Chronoamperometry Measurements

**Figure 6:**
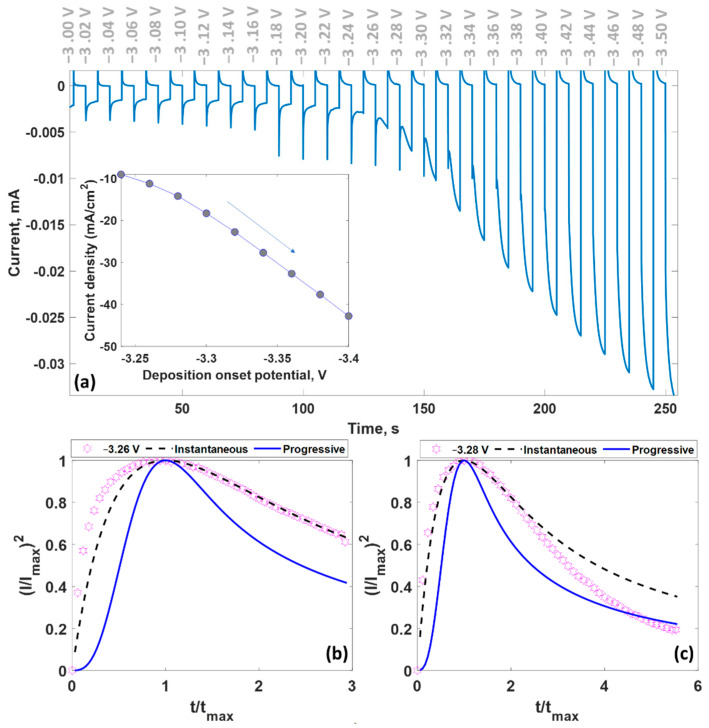
(**a**) Chronoamperometric curve for the electrodeposition of lithium from PC|LiClO_4_ electrolyte on bilayer-patched epitaxial graphene under potential stepping from −3 V to −3.5 V. Inset demonstrates the relationship between the maximum current density and deposition potential. (**b**,**c**) Comparison of the experimental current transient curves registered at different potentials (−3.26 and −3.28 V) with theoretical modeling for instantaneous and progressive nucleation mechanism.

To elucidate the kinetics of lithium, we performed chronoamperometry measurements and recorded the current–time transients during the electrodeposition of lithium on bilayer-patched epitaxial graphene on 4H-SiC. When the electrode is stepped from an initial potential to larger potentials, the current density significantly increases (Figure 6a), which is associated with enlarging surface density of the active lithium species due to the faster nucleation rate. When the deposition potential is shifted toward larger potentials, the maximum current increases (inset in Figure 6a), while the maximum time tends to become shorter (Figure S1, Supporting Information). According to the Scharifker–Hill nucleation model [88], two significant nucleation processes can be distinguished: instantaneous nucleation (formation of the critical nuclei only during the first stages of the Li electrodeposition followed by their eventual growth within predefined diffusion zones) and progressive nucleation (continuous formation of new nuclei during Li electrodeposition process). The mentioned models can be described by using the following equations:

(1) {(jinstjmax)2=1.9542(ttmax)−1{1−exp[−1.2564(ttmax)]}2(jprogjmax)2=1.2254(ttmax)−1{1−exp[−2.3367(ttmax)2]}2

where *t_max_* is the time at which the current ($j_{inst}\;$or $j_{prog}$) reaches the maximum value *j_max_*. The comparison of the theoretical and experimental curves makes it possible to identify the dominating mechanism. The experimental dependence of the dimensionless current density on the dimensionless time is well fit by the expression describing the instantaneous nucleation mechanism (Figure 6b,c). It seems that this mechanism is a common mechanism for the growth of metals (Pb [71], Cu [89], Hg [90], and Li) on epitaxial graphene on 4H-SiC. In fact, only a limited number of active sites are available for metal nuclei formation during the electrodeposition process. Using *t_max_* and *j_max_* values, we then estimated the diffusion coefficient of Li^+^ ions as follows [91]:

(2) $$D=\frac{j_{max\;}^{2}t_{max}}{0.1629\cdot {\left(z\cdot F\cdot C\right)}^{2}}$$

where z is the valency of the metal ion (+1 in the case of monovalent Li species), F is the Faraday constant (96 485 C∙mole^−1^), and C is the reactant concentration. The values of diffusion coefficients range from ~4.7 to 5.6 ×10^−7^ cm^2^∙s^−1^ (also see Table 1), which are comparable to the fast lithium-ion in-plane diffusivity in graphitic carbon (~10^−7^–10^−6^ cm^2^∙s^−1^), but at the same time, they are much higher than the sluggish lithium-ion diffusivity perpendicular to the basal plane of graphene (~10^−11^ cm^2^∙s^−1^) [92]. It is interesting to note that the estimated values of Li diffusivity are also consistent with literature data for the lithium diffusion process in lithium electrolytes (ca. 10^−7^ cm^2^∙s^−1^), indicating an efficient Li ion transport through the SEI [93].

Finally, the saturation nucleation density on the bilayer-patched epitaxial graphene electrode was determined by using the following equation:

(3) $$\;N_{0}=0.065{\left(\frac{1}{8\pi CV_{m}}\right)}^{1/2}{\left(\frac{nFC}{I_{max}t_{max}}\right)}^{2}$$

where *n* is the number of electrons involved and *V*_m_ is the molar volume. From Table 1, it is seen that *N*_0_ demonstrates a generally increasing trend, reaching a value of ~3.0 × 10^6^ cm^−2^ at −3.3 V. This is because the nucleation rate is directly proportional to the potential applied.

Based on the results presented above, it is apparent that the initial kinetics of the lithium species at the bilayer-patched epitaxial graphene surface is mainly regulated by the instantaneous nucleation mechanism. This means that during the early stages of the electrodeposition process, the surface of the BLPMLG/SiC electrode is covered by separated lithium nuclei with discrete diffusion zones. From the practical point of view, this mechanism is ideal to provide fast and repetitive plating and stripping of lithium species, thereby allowing one to avoid/minimize dendritic growth that causes the battery failure. Furthermore, a high nuclei number density implies that the SEI formed for the PC|LiClO_4_ system completely covers the electrode surface, facilitating lithium-ion diffusion. It should be mentioned that in the case of partial coverage of the electrode surface with an SEI, one would expect lower values of the nuclei number density due to deposition directly on the SiC surface. DFT calculations allowed us to obtain atomistic insights into lithium electrodeposition at the initial stages. Particularly, we found that the hollow site is the energetically most favorable site for lithium adsorption on MLG/SiC, AA-BLG/SiC, and AB-BLG/SiC electrodes (Figure S2, Supporting Information). The BSSE-corrected adsorption energies of lithium at all considered electrodes were 0.9468, 1.0075, and 0.9338 eV, respectively. Although the AA-BLG/SiC electrode demonstrates the strongest binding ability with respect to lithium, the formation of AA-stacked bilayer graphene during the graphenization process is less likely than that of AB-stacked (Bernal stacking) bilayer graphene [94]. The similar adsorption energies in the case of MLG/SiC and AA-BLG/SiC electrodes suggests that there is no adsorption preference of lithium on bilayer-patched epitaxial graphene. In other words, lithium can simultaneously occupy adsorption sites of monolayer and bilayer graphene regions. Charge population analysis using Hirshfeld and Voronoi schemes shows that there is a strong charge transfer from lithium to MLG/SiC, AA-BLG/SiC, and AB-BLG/SiC electrodes. Li atoms donate their *s* electrons and become lithium ions. Table 2 summarizes the parameters describing the lithium adsorption onto the electrode surface.

### 3.4. Raman Probing of Lithiated Bilayer-Patched Epitaxial Graphene

**Figure 7:**
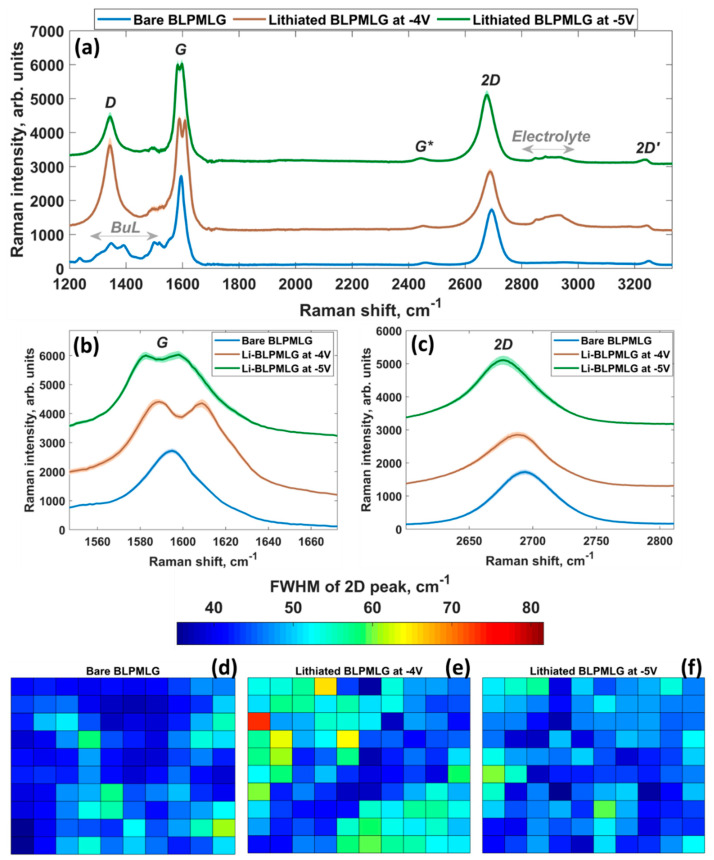
(**a**) Raman spectra of the BLPMLG/SiC electrode before and after lithiation. (**b,c**) Zoomed *G* and 2*D* peak spectral regions for pristine and lithium-intercalated samples, respectively. Maps of FWHM values of 2*D* peak for (**d**) bare BLPMLG/SiC electrode and for electrodes lithiated at (**e**) −4 V and (**f**) −5 V. Each colored box represents one Raman spectrum. 121 Raman spectra per map were analyzed to construct the maps.

To better comprehend the nature of the interaction between lithium and BLPMLG/SiC, we also performed Raman mapping analysis before and after lithium electrodeposition. As mentioned above, the average Raman spectrum of pristine BLPMLG/SiC is characterized by the presence of three most prominent spectral features: buffer-layer-related features ranging from 1000 cm^−1^ to 1600 cm^−1^, *G* peak, and 2*D* peak (Figure 7). It is clearly seen that after lithium electrodeposition at −4 V (duration: 5 min) and −5 V (1 min), Raman spectra of BLPMLG/SiC undergo significant changes. In particular, we notice the disappearance of the buffer layer-related spectral features, which is probably due to the lithium intercalation-induced buffer layer decoupling from SiC followed by its transformation to a graphene layer. All Raman spectra of lithiated samples also exhibit a defect-related *D* peak (see additional details including Figure S3 in Supporting Information), which could be from the generation of *sp*^3^-type defects forming during the lithiation processes, and the wide band ranging from 2800 to 3000 cm^−1^. The latter overlaps with the fingerprint region of C-H stretching modes and can be assigned to the formation of the reduction products of lithium salt [95,96]. The intensity of both bands can be controlled via fine tuning the electrodeposition conditions. These results provide direct evidence of the SEI growth onto the electrode surface during the lithiation process. The interaction between the SEI layer and BLPMLG/SiC can also be responsible for the activation of the initially forbidden *D* mode. Finally, we notice an obvious *G* peak splitting into two components after lithiation (Figure 7b). This phenomenon can be ascribed to the presence of two physically inequivalent graphene layers with different lithium-induced doping levels. Similar effects have been reported for other types of lithiated bilayer graphene systems [97,98,99].

**Figure 8:**
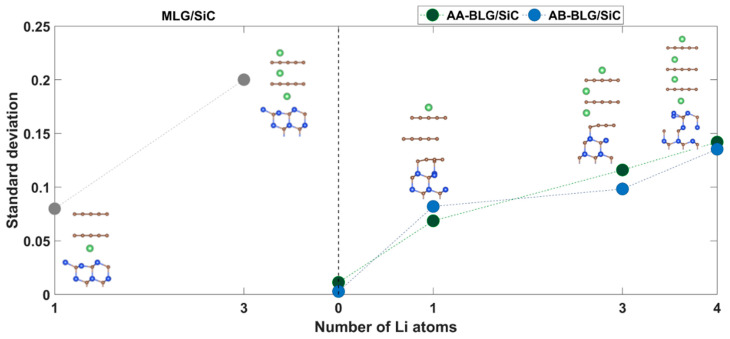
Effect of the number of lithium atoms inserted in epitaxial graphene on the charge redistribution between graphene layers. Here, we initially estimated the total Hirshfeld charge accumulated by a separate graphene layer and then determined the inequivalence of graphene sheets in terms of the standard deviation. The larger the standard deviation, the greater the disparities between graphene layers. The images inside the figure correspond to the optimized structures of different lithiated electrodes. For the sake of simplicity, we demonstrate only a few topmost Si-C bilayers.

The comparison of the FWHM of 2*D* peaks of bare BLPMLG/SiC and lithiated BLPMLG/SiC (Figure 7e,f) shows a 2*D* band broadening caused by lithiation and a significant increase in the number of Raman spectra with a 2*D* band broader than 45 cm^−1^. This is indicative of the lithium intercalation beneath graphene, which is accompanied by the appearance of the additional graphene layer and lithium insertion between layers that causes different electron–phonon interactions manifested as Raman *G*-band splitting. Charge population analysis of different lithium-intercalated electrode cases corroborates this notion (Figure 8) by clearly indicating that the increase in lithiation degree causes an increase in nonequivalent doping of graphene layers. This also explains why we observe larger *G* peak splitting for the sample lithiated at −4 V during 5 min compared to that for the electrode lithiated at −5 V during 1 min (Figure 7d). The former conditions are more beneficial for efficient lithium intercalation than the latter.

**Figure 9:**
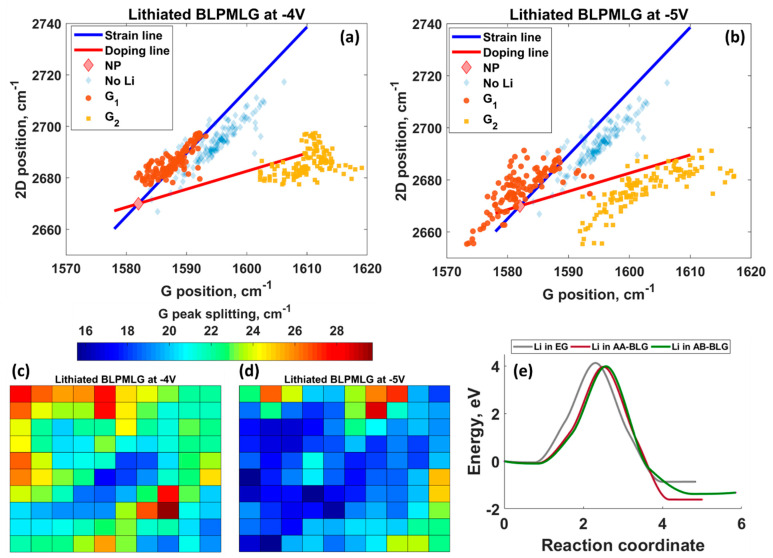
2*D* peak position vs. *G* peak position for bilayer-patched epitaxial graphene after lithiation at −4 V (**a**) and −5 V (**b**). The Raman data for bare bilayer-patched epitaxial graphene are also shown. The reddish diamond designated by NP represents the neutrality point of free-standing graphene, without doping and strain effects. Maps of *G* peak splitting values for electrodes lithiated at (**c**) −4 V and (**d**) −5 V. Each colored box represents one Raman spectrum. 121 Raman spectra per sample were analyzed to construct the maps. (**e**) CI-NEB energy curvatures for lithium penetrating beneath the topmost graphene layer in MLG/SiC, AA-BLG/SiC, and AB-BLG/SiC electrodes.

The comparison of the 2*D* vs. *G* peak dependencies before and after lithiation reveals a significant difference (Figure 9a,b). In contrast to the expected picture for the BLPMLG/SiC sample before lithiation, we observed a completely different picture for the BLPMLG/SiC after lithiation. As a result of the *G* peak splitting, we observed two different statistics, suggesting the presence of graphene layers with different doping degrees. One group of data points, which are related to the lower doping level, scatter along the strain line. Another set of data points corresponds to the highly doped graphene regions. We then plotted the images of the *G* peak splitting value to show the distribution of the splitting over the sample (Figure 9c,d). It is clearly seen that the lithiation at the potential of −4 V leads to more pronounced *G* peak splitting compared to that at −5 V, suggesting an enhanced lithium insertion. To better understand how the lithium insertion into bilayer-patched epitaxial graphene electrodes occurs at initial lithiation stages, we then performed CI-NEB calculations to estimate the energy barriers for lithium penetration beneath the topmost graphene layer in MLG/SiC, AA-BLG/SiC, and AB-BLG/SiC electrodes (Figure 9e, also see Figures S4–S6 in Supporting Information). Interestingly, the energy barriers for AA-BLG/SiC and AB-BLG/SiC electrodes are 3.966 and 3.988 eV, respectively, which are lower than that for the MLG/SiC (4.128 eV). These results indicate that the early-stage intercalation process predominantly occurs through the bilayer graphene, highlighting the important role of bilayer graphene patches in the lithiation of epitaxial graphene.

## 4. Conclusions

In conclusion, we demonstrated the potential of bilayer-patched epitaxial graphene on 4H-SiC as an anode for rechargeable batteries. Tapping-mode AFM and Scanning Capacitance Microscopy techniques, and reflectance and Raman mappings were employed to confirm the co-existence of monolayer and bilayer graphene within one electrode platform. By performing cyclic voltammetry measurements, we found that the anodic peak currents were increased and shifted to less negative voltages from cycle to cycle. We ascribed this effect to the formation of the stable, highly ionically conductive, and uniform SEI at the electrode surface that promotes fast interfacial lithium transport. XPS and XPEEM studies performed by a synchrotron radiation facility showed that lithium at the BLPMLG/SiC electrode surface existed in two chemical states (metallic and oxidized), thereby providing additional evidence of SEI formation. Raman studies brought up another argument in favor of our assumption on SEI growth. Particularly, we attributed the wide phonon band at ~2800 to 3000 cm^−1^ in the Raman spectra of lithiated samples to a fingerprint frequency region of products of the electrolyte reduction and hence SEI. SEI formation was also identified as a possible reason for the activation of the defect-related Raman *D* peak. Chronoamperometry measurements enabled us to elucidate the nature of the early-stage lithium nucleation at the BLPMLG/SiC electrode surface. The dominant role of the instantaneous nucleation mechanism was established. Although DFT calculations did not reveal the preference of the AB-stacked bilayer graphene over monolayer graphene in terms of lithium adsorption energy, we expect that AB-stacked bilayer graphene patches will ensure the favored intercalation channels due to the lower energy barrier for lithium intercalation in respect of the monolayer graphene. Finally, in-depth statistical analysis of the Raman data supported by DFT calculations allowed us to link the Raman *G* peak splitting to the inequivalent doping of graphene layers. The present results gain deep insights into the nature of the lithiation of bilayer-patched epitaxial graphene on 4H-SiC and may boost both the development of the appealing alternative anode materials for next-generation rechargeable batteries and more sophisticated experimentation on epitaxial graphene.

## Figures and Tables

**Table 1:** Summarized parameters from Hills–Scharifker theoretical analysis of the chronoamperometry results.

Potential, V	Time, s	Maximum Current Density, A/cm^2^	Mechanism	Diffusion Coefficient, ×10^−6^ cm^2^∙s^−1^	Nuclei Number Density, ×10^7^ cm^−2^
−3.24	2.6	−0.0090	instantaneous	0.5595	0.0674
−3.26	1.7	−0.0112	instantaneous	0.5651	0.1021
−3.28	0.9	−0.0142	instantaneous	0.4782	0.2279
−3.3	0.6	−0.0183	instantaneous	0.5294	0.3088

**Table 2:** DFT-derived parameters describing lithium adsorption on all considered electrodes. The positive charges on Li atom means that the charge is transferred from Li to electrode surface.

Electrode	Adsorption Energy, eV		Adsorption Height, Å	Charge on Lithium, *e^−^*	
	Uncorrected	BSSE-Corrected		Hirshfeld	Voronoi
MLG/SiC	1.2161	0.9468	1.6939	0.201	0.217
AA-BLG/SiC	1.2802	1.0075	1.6803	0.209	0.224
AB-BLG/SiC	1.2051	0.9338	1.6973	0.198	0.213

## Data Availability

The data that support the findings of this study are available within the article.

## Supplementary Materials

The following supporting information can be downloaded at: https://www.mdpi.com/article/10.3390/nano12132229/s1, Figure S1: The relationship between the maximum time that corresponds to maximum current density, and the deposition potential; Figure S2: (Side and top views) Optimized structures of MLG/SiC, AA-BLG/SiC, and AB-BLG/SiC electrodes with adsorbed Li atom. Blue, green, whitish, and brown balls represent silicon, lithium, hydrogen, and carbon atoms, respectively; Figure S3: Spectral regions corresponding to *D* Raman peak of BLPMLG/SiC lithiated at (a) −4 V and (b) −5 V. Grey dots are experimental data, while the solid dark red curves are the fitting Lorentzian curves. Figure S4: Initial (a), transition (b), and final (c) state structures of lithiated MLG/SiC electrode. Transition state structure was predicted by CI-NEB calculations, while initial and final structures were relaxed using the conventional DFT method. Blue, green, whitish, and brown balls represent silicon, lithium, hydrogen, and carbon atoms, respectively; Figure S5: Initial (a), transition (b), and final (c) state structures of lithiated AA-BLG/SiC electrode. Transition state structure was predicted by CI-NEB calculations, while initial and final structures were relaxed using the conventional DFT method. Blue, green, whitish, and brown balls represent silicon, lithium, hydrogen, and carbon atoms, respectively; Figure S6: Initial (a), transition (b), and final (c) state structures of lithiated AB-BLG/SiC electrode. Transition state structure was predicted by CI-NEB calculations, while initial and final structures were relaxed using the conventional DFT method. Blue, green, whitish, and brown balls represent silicon, lithium, hydrogen, and carbon atoms, respectively.
