# Supplementary material for: Understanding of the Electrochemical Behavior of Lithium at Bilayer-Patched Epitaxial Graphene/4H-SiC
Source: Nanomaterials (Basel). 2022 Jun 29;12(13):2229. doi: 10.3390/nano12132229 (PMC9268403; doi:10.3390/nano12132229)
Supplement: Supplementary file 1 [file nanomaterials-12-02229-s001.zip › nanomaterials-1792577-supplementary.pdf]

nanomaterials

Supplementary Materials

Understanding of the Electrochemical Behavior of Lithium at Bilayer-Patched Epitaxial Graphene/4H-SiC

Ivan Shtepliuk [1,*], Mikhail Vagin [2], Ziyauddin Khan [2], Alexei A. Zakharov [3], Tihomir Iakimov [1], Filippo Giannazzo [4], Ivan G. Ivanov [1] and Rositsa Yakimova [1]

[1] Department of Physics, Chemistry and Biology, Linköping University, SE-58183 Linköping, Sweden; ivan.shtepliuk@liu.se (I.S.); tihomir.iakimov@liu.se (T.I.); ivan.gueorguiev.ivanov@liu.se (I.G.I.); rositsa.yakimova@liu.se (R.Y.)
[2] Laboratory of Organic Electronics, Department of Science and Technology, Linköping University, SE-60174 Norrköping, Sweden; mikhail.vagin@liu.se (M.V.); ziyauddin.khan@liu.se (Z.K.)
[3] MAX IV Laboratory, Lund University, Fotongatan 2, SE-22484 Lund, Sweden; alexei.zakharov@maxiv.lu.se
[4] CNR-IMM, Strada VIII, 5, 95121 Catania, Italy; Filippo.Giannazzo@imm.cnr.it
* Correspondence: ivan.shtepliuk@liu.se; Tel.: +46-766-524-089

Citation: Shtepliuk, I.; Vagin, M.; Khan, Z.; Zakharov, A.A.; Iakimov, T.; Ivanov, I.G.; Yakimova, R. Understanding of the Electrochemical Behavior of Lithium at Bilayer-Patched Epitaxial Graphene/4H-SiC. *Nanomaterials* **2022**, *12*, 2229. https://doi.org/10.3390/nano12132229

Academic Editors: Alessandro Molle and Emiliano Bonera

Received: 13 June 2022
Accepted: 28 June 2022
Published: 29 June 2022

Publisher's Note: MDPI stays neutral with regard to jurisdictional claims in published maps and institutional affiliations.

Copyright: © 2022 by the authors. Licensee MDPI, Basel, Switzerland. This article is an open access article distributed under the terms and conditions of the Creative Commons Attribution (CC BY) license (https://creativecommons.org/licenses/by/4.0/).

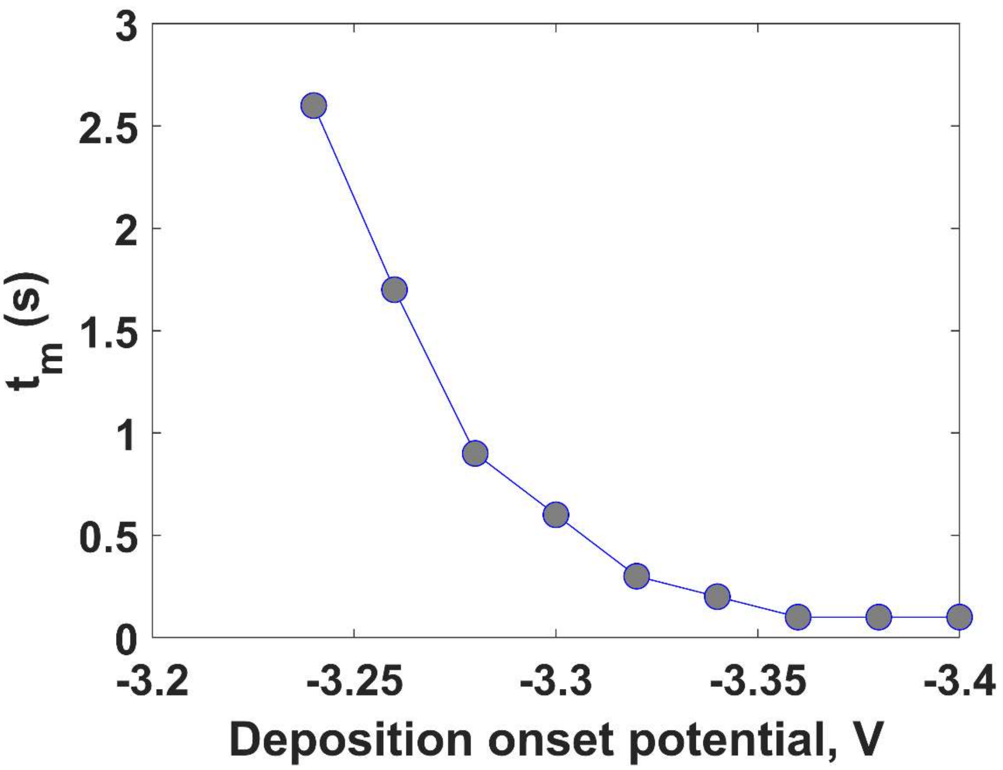

Figure S1. The relationship between the maximum time that corresponds to maximum current density, and the deposition potential.

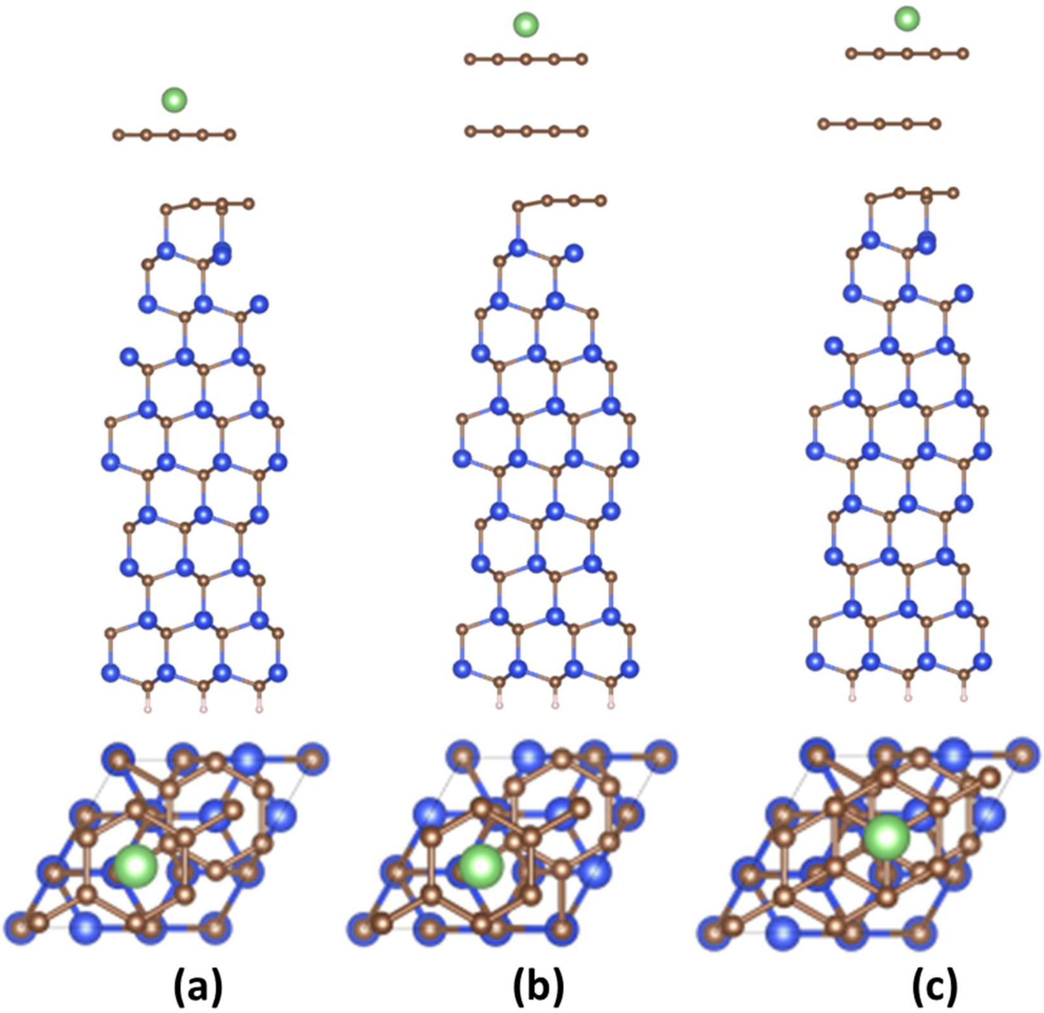

Figure S2. (Side and top views) Optimized structures of (**a**) MLG/SiC, (**b**) AA-BLG/SiC, and (**c**) AB-BLG/SiC electrodes with adsorbed Li atom. Blue, green, whitish, and brown balls represent silicon, lithium, hydrogen, and carbon atoms, respectively.

D Raman peak analysis

The *D* Raman mode region can be well fitted by a single Lorentzian peak with fitting errors as low as 3.158% and 3.015% for samples lithiated at −4 V and −5 V, respectively. The corresponding goodness of fit (R^2) is predicted to be 0.988 for both cases (see Figure S2, Supporting Information). As we observed *G* peak splitting into two components, we should estimate two different amplitude ratios: D/G_1 and D/G_2. As these ratios for BLPMLG/SiC lithiated at −4V are larger than those for that lithiated at −5V (0.90 and 1.15 vs. 0.51 and 0.49, respectively), one can only conclude that the latter lithiation conditions result in reduced defectiveness of BLPMLG/SiC.

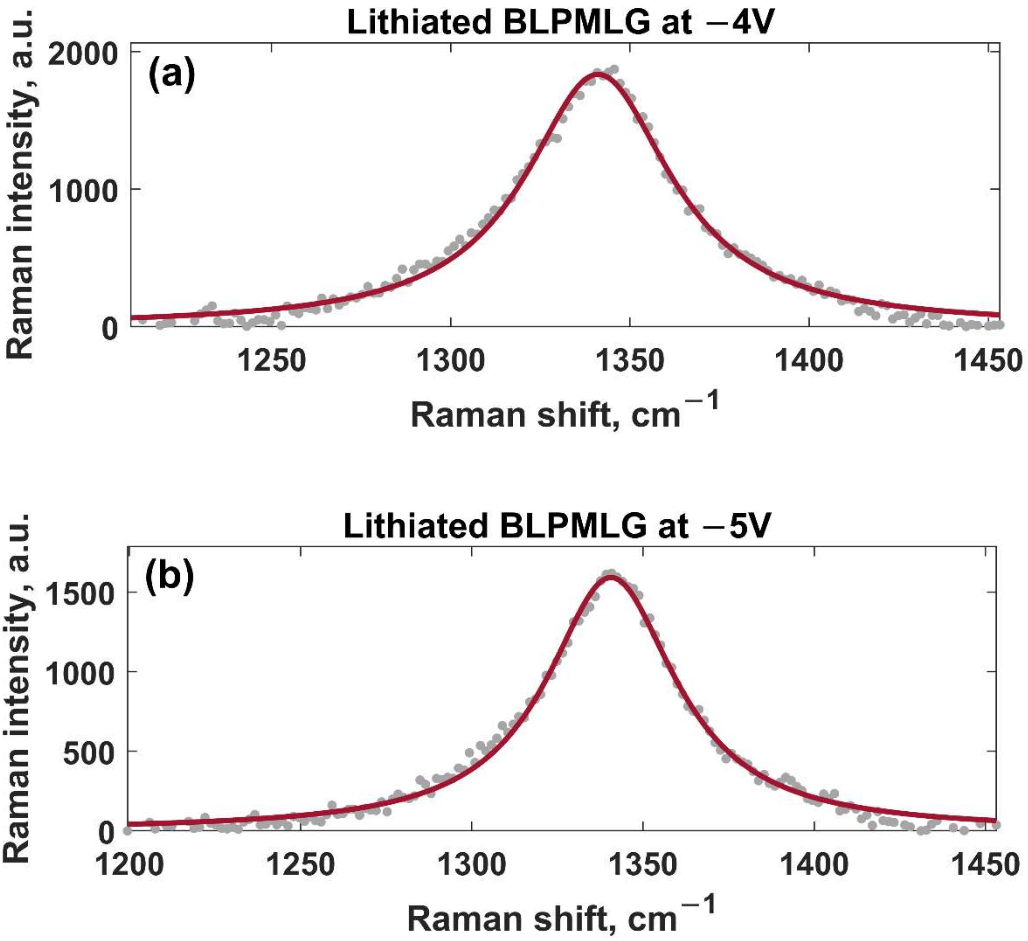

Figure S3. Spectral regions corresponding to *D* Raman peak of BLPMLG/SiC lithiated at (**a**) −4 V and (**b**) −5 V. Grey dots are experimental data, while the solid dark red curves are the fitting Lorentzian curves.

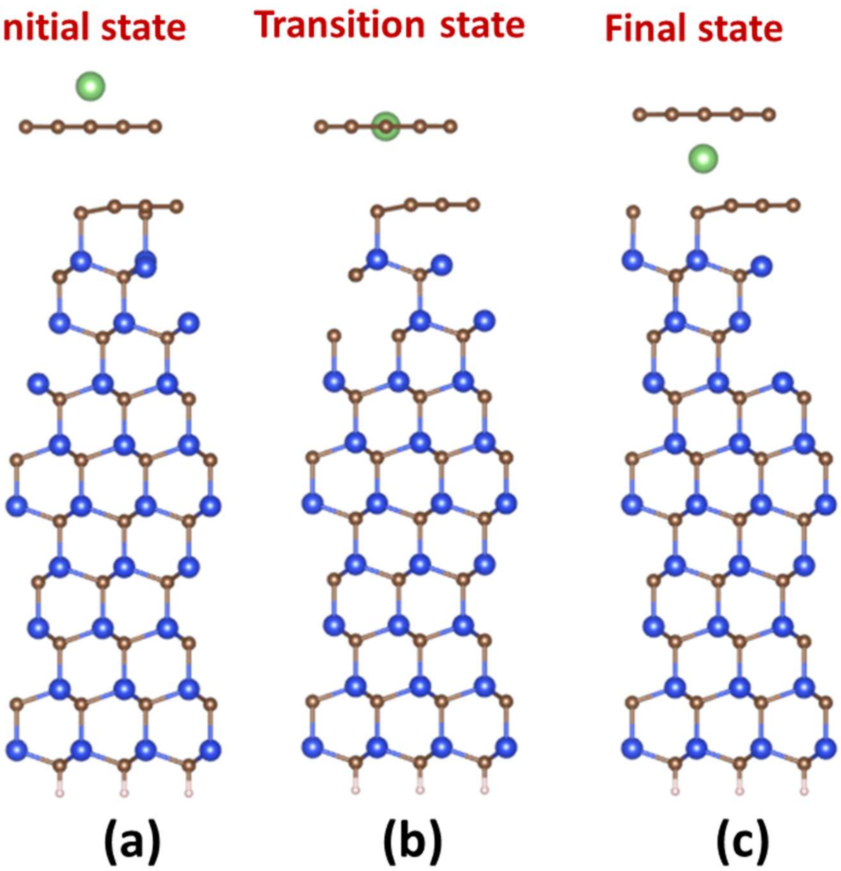

Figure S4. Initial (**a**), transition (**b**), and final (**c**) state structures of lithiated MLG/SiC electrode. Transition state structure was predicted by CI-NEB calculations, while initial and final structures were relaxed using the conventional DFT method. Blue, green, whitish, and brown balls represent silicon, lithium, hydrogen, and carbon atoms, respectively.

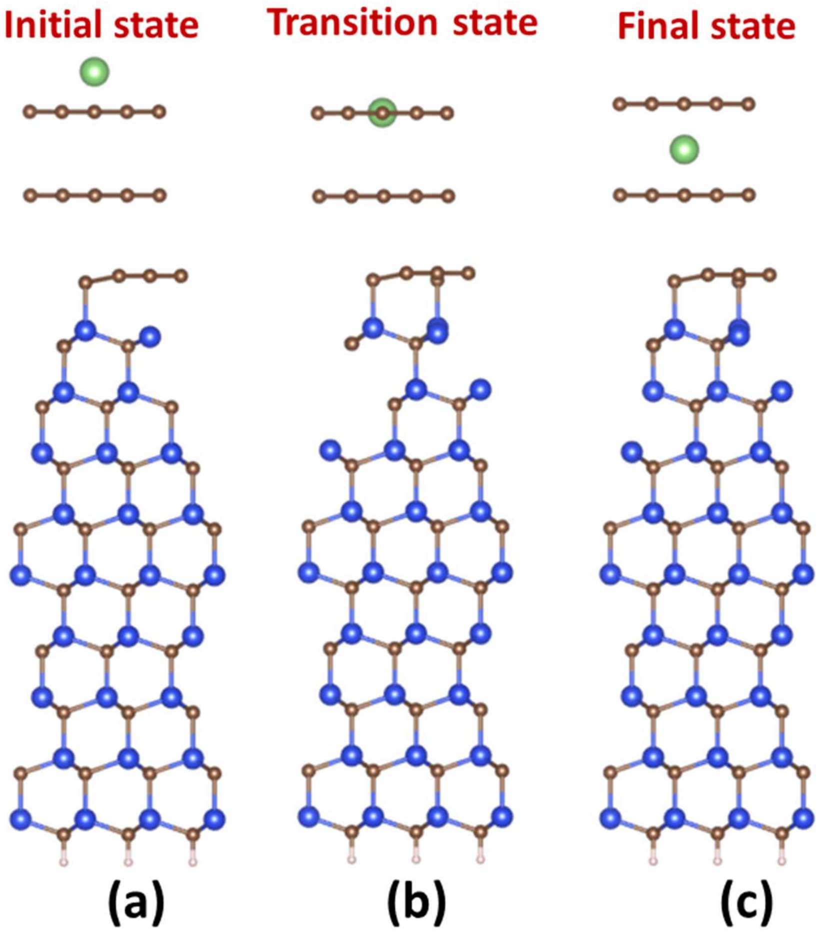

Figure S5. Initial (**a**), transition (**b**), and final (**c**) state structures of lithiated AA-BLG/SiC electrode. Transition state structure was predicted by CI-NEB calculations, while initial and final structures were relaxed using the conventional DFT method. Blue, green, whitish, and brown balls represent silicon, lithium, hydrogen, and carbon atoms, respectively.

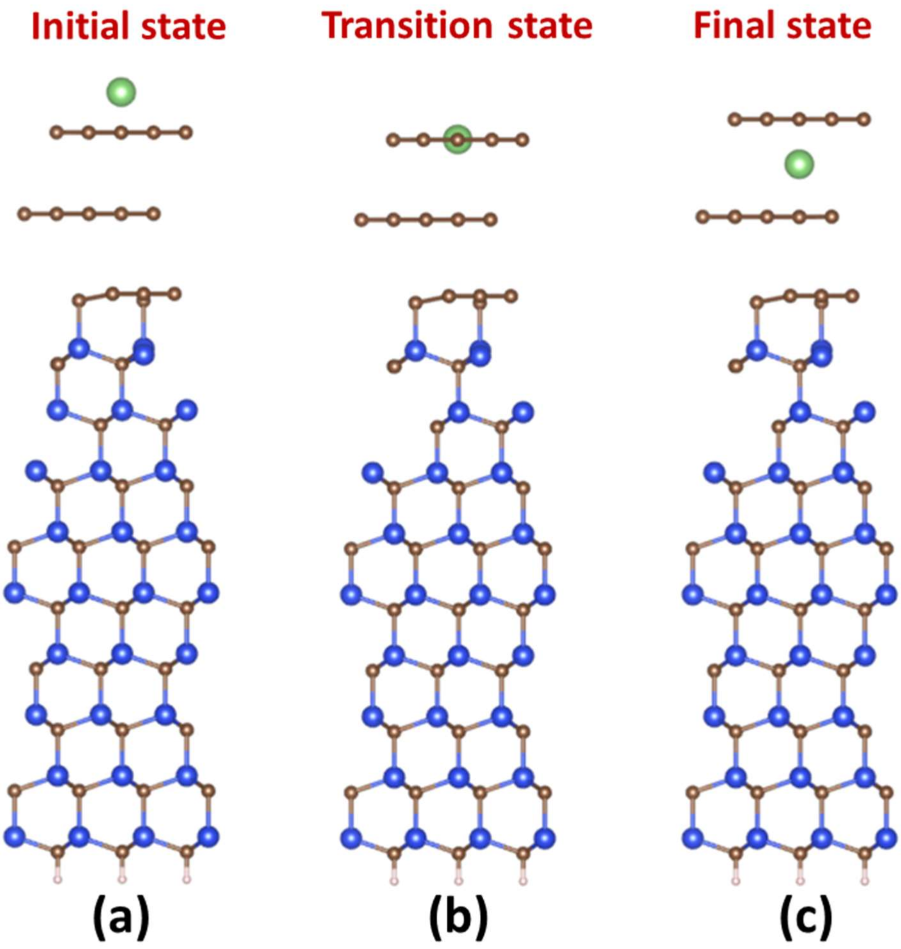

Figure S6. Initial (**a**), transition (**b**), and final (**c**) state structures of lithiated AB-BLG/SiC electrode. Transition state structure was predicted by CI-NEB calculations, while initial and final structures were relaxed using the conventional DFT method. Blue, green, whitish, and brown balls represent silicon, lithium, hydrogen, and carbon atoms, respectively.
